# A Tissue Biomarker Panel Predicting Systemic Progression after PSA Recurrence Post-Definitive Prostate Cancer Therapy

**DOI:** 10.1371/journal.pone.0002318

**Published:** 2008-05-28

**Authors:** Tohru Nakagawa, Thomas M. Kollmeyer, Bruce W. Morlan, S. Keith Anderson, Eric J. Bergstralh, Brian J. Davis, Yan W. Asmann, George G. Klee, Karla V. Ballman, Robert B. Jenkins

**Affiliations:** 1 Department of Laboratory Medicine and Pathology, Mayo Clinic, Rochester, Minnesota, United States of America; 2 Department of Health Sciences Research, Mayo Clinic, Rochester, Minnesota, United States of America; 3 Department of Radiation Oncology, Mayo Clinic, Rochester, Minnesota, United States of America; University of Minnesota, United States of America

## Abstract

**Background:**

Many men develop a rising PSA after initial therapy for prostate cancer. While some of these men will develop a local or metastatic recurrence that warrants further therapy, others will have no evidence of disease progression. We hypothesized that an expression biomarker panel can predict which men with a rising PSA would benefit from further therapy.

**Methodology/Principal Findings:**

A case-control design was used to test the association of gene expression with outcome. Systemic (SYS) progression cases were men post-prostatectomy who developed systemic progression within 5 years after PSA recurrence. PSA progression controls were matched men post-prostatectomy with PSA recurrence but no evidence of clinical progression within 5 years. Using expression arrays optimized for paraffin-embedded tissue RNA, 1021 cancer-related genes were evaluated–including 570 genes implicated in prostate cancer progression. Genes from 8 previously reported marker panels were included. A systemic progression model containing 17 genes was developed. This model generated an AUC of 0.88 (95% CI: 0.84–0.92). Similar AUCs were generated using 3 previously reported panels. In secondary analyses, the model predicted the endpoints of prostate cancer death (in SYS cases) and systemic progression beyond 5 years (in PSA controls) with hazard ratios 2.5 and 4.7, respectively (log-rank p-values of 0.0007 and 0.0005). Genes mapped to 8q24 were significantly enriched in the model.

**Conclusions/Significance:**

Specific gene expression patterns are significantly associated with systemic progression after PSA recurrence. The measurement of gene expression pattern may be useful for determining which men may benefit from additional therapy after PSA recurrence.

## Introduction

The majority of men with prostate cancer are now diagnosed with cancers that have a low risk of cause-specific mortality [1]. These men are usually treated with radical retropubic prostatectomy (RRP), external beam radiotherapy, or interstitial brachytherapy and are then followed by regular serum PSA evaluations. Over the next 5 to 10 year period, 15–30% of these men will develop a rising PSA [2]–[6], defining a rapidly growing population of major clinical and public health significance. Of this PSA relapse group some men will have local recurrence or have clinically-detectable metastasis, but many will have no other evidence of recurrent prostate cancer other than the rising PSA. The PSA “doubling time” has been identified as a potential surrogate for cause-specific mortality, and is used by some clinicians to determine which men with PSA relapse deserve adjuvant hormonal ablation, local radiation therapy, or simple observation [4]–[6]. Biomarkers that predict which of these men would benefit from any additional therapy are needed.

Large scale gene expression studies of prostate cancers of different grade and stage have been performed by several groups [7]–[16]. These expression studies have utilized arrays containing probe sets of up to 35,000 genes. While these studies are important for biomarker discovery, several difficulties preclude their translation into a clincial setting. First, it is likely that smaller panels will be used clinically. Second, because the previous studies required frozen material, the number of specimens analyzed was limited. Third, since adverse clinical events in prostate cancer patients require lengthy followup, the testing methods must be applicable to archival paraffin-embedded material. Finally, none of the previous studies was focused on the development of a biomarker panel to predict prostate cancer systemic progression in the setting of PSA recurrence.

Using the Mayo Clinic Radical Retropubic Prostatectomy (RRP) Registry, we designed a nested case-control study to test the hypothesis that a limited set of RNA expression biomarkers can predict which men with a rising PSA post-RRP might benefit from additional clinical intervention. The Illumina DASL™ expression microarray platform was selected as the biomarker measurement method, because it measures the expression of gene targets using paraffin tissues [17]–[19]. Using expression data from the literature and derived from our own research program we developed a limited set of expression markers that would likely be altered in association with prostate cancer progression. The panel also included expression biomarkers from several other previously published panels that are associated with surrogates (high Gleason Score, high pathologic stage, or metastatic disease) for prostate cancer aggressiveness [12]–[16].

We report that the array-based measurements showed excellent correlation with quantitative RT-PCR measurements of paraffin-derived RNAs. We also report excellent intra-array, inter-array and within-gene reproducibility. We then describe the testing and validation of a gene expression tissue biomarker panel for the prediction of prostate cancer systemic progression following a rising PSA after radical prostatectomy. We compare the performance of our panel with other previously published panels. Finally, we show that the overexpression of genes mapped to chromosome band 8q24 is associated with prostate cancer systemic progression.

## Methods

### Gene Selection and Array Design for the DASL™ Assay

#### Two Illumina DASL expression microarrays were utilized for the experiments

The standard commercially available Illumina DASL expression microarray (Cancer Panel™ v1) containing 502 oncogenes, tumor suppressor genes and genes in their associated pathways. Seventy-eight of the targets on the commercial array have been associated with prostate cancer progression.

A custom Illumina DASL™ expression microarray containing 526 gene targets for RNAs, including genes whose expression is altered in association with prostate cancer progression. Probes for the custom DASL® panel were designed and synthesized by Illumina, Inc. (San Diego, CA).

Four different sets of prostate cancer aggressiveness genes were included in the study (if the genes were not present on the Cancer Panel v1 array, they were included in the design of the custom array):

1. Markers of prostate cancer aggressiveness identified by a Mayo/University of Minnesota Partnership [20]: The expression profiles of 100 laser-capture microdissected prostate cancer lesions and matched normal and BPH control lesions were analyzed using Affymetrix HG-U133 Plus 2.0 microarrays. Ranked lists of significantly over- and under-expressed genes comparing 10 Gleason 5 and 7 metastatic lesions to 31 Gleason 3 cancer lesions were generated. The top 500 genes on this list were compared to lists generated from prior expression microarray studies and other marker studies of prostate cancer (see 2–4 next). After this analysis there was space for 204 novel targets with potential association with aggressive prostate cancer on the custom array.
2. Markers associated with prostate cancer aggressiveness from publicly available expression microarray datasets (e.g. EZH2, AMACR, hepsin, PRLz, PRL3): When we designed the array sufficiently large datasets from 9 prior microarray studies of prostate cancer of varying grades and metastatic potential [7]–[15] were available from the Oncomine internet site [21], [22], www.oncomine.org. From ordered lists of these data we selected 32 genes for inclusion on the array.
3. Previously published markers associated with prostate cancer aggressiveness (e.g. PSMA, PSCA, Cav-1): Expression microarray data has also been published. This literature was evaluated for additional tissue biomarkers. For example, at the time of array design we were able to identify 13 high quality expression microarray studies of prostate cancer aggressiveness (See Tables S1 and S2 for full reference list). In addition, among the 13 reports, 5 papers presented 8 expression biomarker panels to predict prostate cancer aggressiveness [12]–[16]. When appropriate probes suitable for the DASL chemistry could be designed for these panels they were included on the custom array. We also identified 12 articles reviewing genes associated with prostate cancer. These criteria resulted in the selection of 150 genes.
4. Markers derived from Mayo SPORE research (including genes and ESTs mapped to 8q24). Ninety-three additional biomarkers were identified (see Tables S1 and S2).

The custom array also included probe sets for 45 genes that were not expected to differ between case and control groups based on Mayo/University of Minnesota Partnership data. Thirty-eight of these genes were also present on the commercial array (see Tables S1 and S2).

After enumerating the potentially prostate cancer relevant genes on the commercially available cancer panel 570 potentially prostate cancer relevant genes and 451 other cancer-related genes were evaluated across both arrays.

### Design of Nested Case-Control Study

For this study we sampled individuals from the Mayo Clinic RRP Registry. The registry consists of a population of men who received prostatectomy as their first treatment for prostate cancer at the Mayo Clinic (For a current description and use of the registry; see reference [23]). As systemic progression is relatively infrequent, we designed a case-control study nested within a cohort of men with a rising PSA. Between 1987–2001, inclusive, 9,989 previously-untreated men had RRP at Mayo. On follow-up, 2,131 developed a rising PSA (>30 days after RRP) in the absence of concurrent clinical recurrence. PSA rise was defined as a follow-up PSA > = 0.20 ng/ml, with the next PSA at least 0.05 ng/ml higher or the initiation of treatment for PSA recurrence (for patients whose follow-up PSA was high enough to warrant treatment). This group of 2,131 men comprises the underlying cohort from which SYS cases and PSA controls were selected.

Within 5 years of PSA rise, 213 men developed systemic progression (SYS cases), defined as a positive bone scan or CT scan. Of these, 100 men succumbed to a prostate cancer-specific death, 37 died from other causes and 76 remain at risk.

PSA recurrence controls (213) were selected from those men without systemic progression within 5 years after the PSA rise and were matched (1:1) on birth year, calendar year of PSA rise and initial diagnostic pathologic Gleason score (< = 6, 7+). Twenty of these men developed systemic progression greater than 5 years after initial PSA rise and 9 succumbed to a prostate cancer-specific death.

A set of 213 No Evidence of Disease (NED) Progression controls were also selected from the Mayo Clinic RRP Registry of 9,989 men and used for some comparisons. These controls had RRP from 1987–1998 with no evidence of PSA rise within 7 years of RRP. The median (25^th^, 75^th^ percentile) follow-up from RRP was 11.3 (9.3, 13.8) years. The NED controls were matched to the systemic progression cases on birth-year, calendar year of RRP and initial diagnostic Gleason Score. Computerized optimal matching was performed to minimize the total “distance” between cases and controls in terms of the sum of the absolute difference in the matching factors [24].

The current study was approved by the Institutional Review Board of Mayo Clinic.

### Block Identification, RNA Isolation, and Expression Analysis

The list of 639 cases and controls was randomized. An attempt was made to identify all available blocks (including apparently normal and abnormal lymph nodes) from the randomized list of 639 eligible cases and controls. Maintaining the randomization, each available block was assessed for tissue content by pathology review and the block containing the dominant Gleason pattern cancer was selected for RNA isolation.

Four freshly cut 10μm sections of FFPE tissue were deparaffinized and the Gleason dominant cancer focus was macrodissected. RNA was extracted using the High Pure RNA Paraffin Kit from Roche (Indianapolis, IN). RNA was quantified using ND-1000 spectrophotometer from NanoDrop Technologies (Wilmington, DE). The RNAs, including intra-plate and inter-plate replicates, were distributed on 96-well plates in the randomized order for DASL analysis.

RNA samples were processed, hybridized to Sentrix Universal 96-Arrays, scanned using BeadArray Reader, and data initially processed in BeadStudio according to the manufacturer's instructions. Microarray data is available from the GEO database (http://www.ncbi.nlm.nih.gov/geo/ accession number GSE10645).

To evaluate the accuracy of the gene expression levels defined by the DASL technology, we performed quantitative SYBR Green RT-PCR reactions for 9 selected “target” genes (CDH1, MUC1, VEGF, IGFBP3, ERG, TPD52, YWHAZ, FAM13C1, and PAGE4) and 4 commonly-used endogeneous control genes (GAPDH, B2M, PPIA and RPL13a) in 384-well plates, with the use of Prism 7900HT instruments (Applied Biosystems, Foster City, CA). 210 RNA samples with abundant RNA from the group of total 639 patients were analyzed. Because of RNA shortage, only 77 samples were analyzed for PAGE4. mRNA was reverse-transcribed with SuperScript III First Strand Synthesis SuperMix (Invitrogen, Carlsbad, CA) using random hexamers. For each of the nine genes studied, the cycle threshold (Ct) was determined in triplicate and the expression was normalized relative to the set of four reference genes.

### Pathology Review

The Gleason score in the Mayo Clinic RRP Registry was defined as the initial Gleason score. Since there have been changes in pathologic interpretation of the Gleason score over time, a single pathologist (JCC) reviewed the Gleason score of each of the blocks selected for expression analysis. This clinical variable was defined as the revised Gleason score.

### Statistical Methodology

Collection of gene expression data was attempted for the 623 patients as described in Results. Of these, there were 596 (n_SYS_ = 200, n_PSA_ = 201, n_NED_ = 195) patients for whom data was collected, the rest having failed one or both expression panels as described in Results. To assure selection of similar training and validation sets, 100 case-control-control cohorts comprised of 133 randomly chosen SYS patients (two-thirds of 200 for training) along with their matched PSA and NED controls were selected as a proposed training set. The remaining cases and controls were treated as a proposed validation set. The clinical variables were tested for independence between the proposed training and validation sets separately within the SYS cases and the PSA controls. Discrete clinical factors (pathologic stage, hormonal treatment adjuvant to RRP, radiation treatment adjuvant to RRP, hormonal treatment adjuvant to PSA recurrence, and radiation therapy adjuvant to PSA recurrence) were tested using Chi–square analysis. Continuous clinical variables (Gleason score (revised), age at PSA recurrence, first rising PSA value, second rising PSA value, and PSA slope) were tested using Wilcoxon rank sum. Six of the one hundred randomly sampled sets failed to show dependency for any of the clinical variables at the 0.2 level, and the first of these was chosen as the training set: 391 patients (n_SYS_ = 133, n_PSA_ = 133, n_NED_ = 125). This reserved 205 patients for the validation set (n_SYS_ = 67, n_PSA_ = 68, n_NED_ = 70).

#### The raw data from BeadStudio was normalized using cyclic loess (fastlo) [25]

The training data were analyzed using random forests [26] using R Version 2.3.1 (http://www.r-project.org) and randomForest version 4.5–16 (http://stat-www.berkeley.edu/users/breiman/RandomForests). The data were analyzed by panel (Cancer, Custom and Merged, where Merged was the Cancer and Custom data treated as a single array). By testing the *ntree* parameter of the randomForest function we determined that 4000 random forests were sufficient to generate a stable list of markers. The top markers as sorted for significance by the randomForest program were combined with various combinations of clinical variables using logistic regression R program (glm() with family = binary (a logistic model), where glm refers to generalized linear model). The resulting scoring function was then analyzed using Receiver Operating Characteristic (ROC) methods and the cut-off was chosen that assumed an equal penalty for false positives and false negatives. A review of the models permitted a subset of markers to be identified, and a subset of supporting clinical data identified. The number of features in the model was determined by leave 1/3 out Monte Carlo Cross Validation (MCCV) using 100 iterations. The number of features was selected to maximize AUC and minimize random variation in the model. The final model was then applied to the 391 patient training set and the reserved 205 patient validation set. For comparison, other previously reported gene expression models were also tested against the training and validation sets [12]–[16].

We compared the previously reported models for their classification of patients into the known PSA recurrence control and SYS progression case groups. We used the Cramér's V-statistic [27] to compare models.

## Results

### Study Design/Paraffin Block Recovery/RNA Isolation and Expression Panel Success

Briefly, a nested case-control study was performed using the large, well-defined cohort of men with rising PSA following RRP(Figure S1). SYS cases were 213 men who developed systemic progression between 90 days and 5.0 years following the PSA rise. PSA controls were a random sample of 213 men who were 5 years post-RRP with PSA recurrence but with no evidence of further clinical progression. NED controls were a random sample of 213 men who were 7 years post-RRP without PSA rise (the comparison of PSA controls with NED controls-will be presented in a subsequent paper). SYS cases and PSA controls were matched (1:1) for birth year, calendar year of PSA rise, and initial pathologic Gleason score (< = 6 vs. > = 7). The list of eligible cases and controls was randomizeed for the blind ascertainment of blocks, isolation of RNA and performance of the expression array experiments.

Table 1 summarizes the distribution of clinical parameters between the SYS cases and the PSA and NED control groups. There was no significant difference between the groups for the matching variables (there was no significant difference in initial diagnostic Gleason score when the < = 6 and >7 groups-the matching criteria-were compared). Comparison of the initial diagnostic Gleason score to the revised Gleason scores revealed that Gleason scores have increased over time. In addition, the proportion of Gleason 8–10 tumors increased comparing NED controls to PSA controls, and PSA controls to SYS cases. The revised Gleason score was used in all the biomarker modeling.

**Table 1:** Systemic progression (SYS) Case and PSA recurrence (PSA) and no evidence of disease (NED) control patient demographics

	Progression group			p-value	
	NED controls	PSA controls	SYS cases	NED vs. PSA	PSA vs. SYS
**Year of Surgery**				0.707	0.592
N	213	213	213		
Median	1992	1992	1992		
Q1, Q3	1989, 1995	1990, 1995	1989, 1995		
**Age at RRP**				0.682	0.496
N	213	213	213		
Median	67	67	67		
Q1, Q3	61, 70	61, 70	61, 70		
**PSA at RRP**				0.001	0.957
N	205	208	204		
Median	8.1	10.5	10.6		
Q1, Q3	5.1, 13.1	6.4, 21.4	6.5, 20.7		
**Gleason score, original**				0.411	0.024
Missing	12	6	14		
< = 6	45 (22.4%)	48 (23.2%)	46 (23.1%)		
7	139 (69.2%)	129 (62.3%)	94 (47.2%)		
8–10	17 (8.5%)	30 (14.5%)	59 (29.6%)		
**Gleason score, revised**				0.002	<0.001
Missing	8	2	6		
< = 6	50 (24.4%)	32 (15.2%)	8 (3.9%)		
7	114 (55.6%)	113 (53.6%)	75 (36.2%)		
8–10	41 (20.0%)	66 (31.3%)	124 (59.9%)		
**Pathologic stage**				0.138	<0.001
T2N0	118 (55.4%)	95 (44.6%)	59 (27.7%)		
T3aN0	43 (20.2%)	53 (24.9%)	47 (22.1%)		
T3bN0	21 (9.9%)	54 (25.4%)	56 (26.3%)		
T3xN+	31 (14.6%)	11 (5.2%)	51 (23.9%)		
**Ploidy**				0.525	0.001
Missing	13	9	1		
Diploid	136 (68.0%)	128 (62.7%)	97 (45.8%)		
Tetraploid	53 (26.5%)	61 (29.9%)	84 (39.6%)		
Aneuploid	11 (5.5%)	15 (7.4%)	31 (14.6%)		
**Age at PSA recurrence**				NA	0.558
N		213	213		
Median		69.1	69.6		
Q1, Q3		64.2, 73.4	64.7, 73.8		

All paraffin-embedded blocks from eligible men were identified and each block was surveyed for the tissue present (primary and secondary Gleason cancer regions, normal and metastatic lymph nodes, etc.). We macrodissected the dominant Gleason pattern region and attempted to isolate RNA. Illumina Cancer Panel™ and custom prostate cancer panel DASL array analyses were then performed on all RNA specimens. The Methods section and Tables S1 & S2 describe the composition of the Cancer Panel and the design of the custom panel.

Table 2 summarizes the final block availability, the RNA isolation success rate and the success rates of the expression array analyses. Of the 639 eligible patients, blocks were available on 623 (97.5%). RNA was isolated and DASL assays successfully performed on a high proportion of patients/specimens: usable RNA was prepared from all 623 blocks, and the Cancer Panel and custom panel DASL arrays were both successful (after repeating some specimens–see below) on 596 RNA specimens (95.7% of RNAs; 93.3% of design patients). Only 9 (1.4%) RNA specimens failed both panels. The primary reason for these failures was poor RNA quality–as measured by qRT-PCR of the RPL13a gene expression [19]. Of the 1246 initial samples run on both panels, 87 (7.0%) specimens failed. Those specimens for which there was residual RNA were repeated with a success rate of 77.2% (61 of 79 samples).

**Table 2:** Availability of blocks, RNA isolation success and DASL assay success

	Progression Case/Control Group			
	None	PSA	Systemic	Total
Design Number	213	213	213	639
Blocks Available	205	211	207	623 (97.5%)
Usable RNA	205	211	207	623 (100%)
Evaluable Data, Both DASL Panels	195	201	200	596 (95.7%)
Evaluable Data,	3	5	2	10 (1.6%)
Evaluable Data,	2	3	3	8 (1.3%)
Failed Both Panels	5	2	2	9 (1.4%)

### Expression Analysis Reproducibility

Replicate analysis results (Figure S2), RT-PCR comparisons (Figure S3) and inter- and intra-panel gene expression comparisons are described in Results S1.

### Specific Gene Expression Results Comparing the Systemic Progression Cohorts with the PSA Recurrence and No Evidence of Progression Cohorts

#### Univariate Analyses by gene

**Figure 1:**
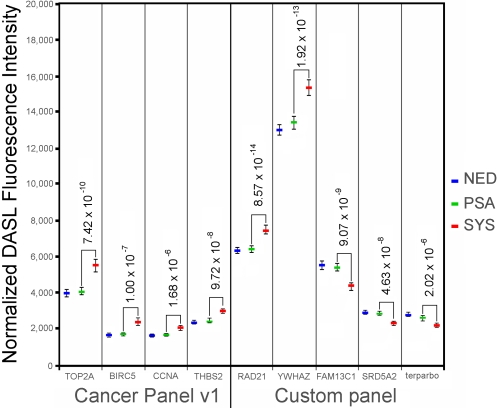
Nine genes with significantly different expression in cases with systemic disease progression (SYS) versus controls with PSA recurrence (PSA). P-values (t-test) for the SYS case/PSA control comparison are shown. Controls with no evidence of disease recurrence (NED) are also included.

Because the DASL assay appeared to generate precise and reproducible results, the array data was examined for genes whose expression was significantly altered when the SYS cases were compared with the PSA controls. For this initial analysis, the DASL gene expression value was determined to be the average of up-to-three probes for each gene on each array. Upon univariate analysis (two-tail t-test) of the probe-averaged and fastlo normalized data [25], 68 genes were highly significantly over- or under-expressed in the SYS cases versus PSA controls (p<9.73×10^−7^, Bonferroni correction for p<0.001) (Table 3). One hundred twenty-six genes were significantly over- or under-expressed in the SYS cases versus the PSA controls (p<4.86×10^−5^, Bonferroni correction for p<0.05). Table S3 provides the complete gene list ordered by p-value. Figure 1 illustrates nine genes with significantly different expression in the SYS cases and PSA controls.

**Table 3:** Top 68 genes highly significantly correlated with prostate cancer systemic progression (p<0.001; with Bonferroni correction p<9.73E-07)

			DASL fast-lo Normalized Expression Value			
rank	Gene Symbol	Gene ID*	Systemic Progression	PSA Recurrence	SYS to PSA Fold Change	SYS to PSA p-value**
1	RAD21***	NM_006265	7587	6409	1.18	8.57E-14
2	YWHAZ	NM_145690	15625	13417	1.16	1.92E-13
3	TAF2***	NM_003184	3144	2681	1.17	6.99E-13
4	SLC44A1	NM_080546	4669	4022	1.16	2.74E-12
5	IGFBP3	NM_000598	4815	3782	1.27	3.75E-12
6	RHOA	NM_001664	15859	14542	1.09	1.22E-11
7	MTPN	NM_145808	7646	6840	1.12	1.69E-11
8	BUB1	NM_001211	1257	957	1.31	2.07E-11
9	TUBB	NM_178014	17412	15659	1.11	6.52E-11
10	CHRAC1***	NM_017444	3905	3233	1.21	6.74E-11
11	HPRT1	NM_000194	3613	3179	1.14	8.19E-11
12	SEC14L1	NM_003003	7248	6185	1.17	8.20E-11
13	SOD1	NM_000454	17412	16043	1.09	1.30E-10
14	ENY2	NM_020189	7597	6493	1.17	2.04E-10
15	CCNB1	NM_031966	1871	1342	1.39	3.65E-10
16	INHBA	NM_002192	4859	3732	1.30	5.18E-10
17	TOP2A	NM_001067	5550	4123	1.35	7.42E-10
18	ATP5J	NM_001003703	13145	11517	1.14	1.75E-09
19	C8orf53***	NM_032334	7373	6444	1.14	1.88E-09
20	EIF3S3***	NM_003756	11946	10798	1.11	1.98E-09
21	EIF2C2***	NM_012154	5908	5338	1.11	2.12E-09
22	CDKN3	NM_005192	1562	1229	1.27	2.32E-09
23	TPX2	NM_012112	1193	861	1.39	2.64E-09
24	GLRX2	NM_197962	4154	3319	1.25	3.13E-09
25	CTHRC1	NM_138455	3136	2480	1.26	3.83E-09
26	KIAA0196***	NM_014846	5530	4945	1.12	4.12E-09
27	DHX9	NM_030588	7067	6607	1.07	5.02E-09
28	FAM13C1	NM_001001971	4448	5416	0.82	9.07E-09
29	CSTB	NM_000100	16424	15379	1.07	1.57E-08
30	SESN3.a	SESN3.a	8467	6811	1.24	1.99E-08
31	SQLE***	NM_003129	2282	1832	1.25	2.43E-08
32	IMMT	NM_006839	4683	4190	1.12	2.43E-08
33	MKI67	NM_002417	4204	3261	1.29	2.91E-08
34	MRPL13***	NM_014078	5051	4158	1.21	3.80E-08
35	SRD5A2	NM_000348	2318	2795	0.83	4.63E-08
36	EZH2	NM_004456	3806	3257	1.17	4.76E-08
37	F2R	NM_001992	3856	3203	1.20	5.61E-08
38	SH3RF2.a	SH3RF2	1394	1705	0.82	6.48E-08
39	ZNF313	NM_018683	9542	8766	1.09	7.14E-08
40	SDHC	NM_001035511	2363	2082	1.14	7.35E-08
41	PGK1	NM_000291	2313	2001	1.16	7.84E-08
42	GNPTAB	NM_024312	5427	4587	1.18	9.04E-08
43	meelar.d	meelar.d	2566	3478	0.74	9.59E-08
44	THBS2	NM_003247	3047	2458	1.24	9.72E-08
45	BIRC5	NM_001168	2451	1802	1.36	1.00E-07
46	POSTN	NM_006475	7210	5812	1.24	1.02E-07
47	GNB1	NM_002074	12350	11206	1.10	1.20E-07
48	FAM49B***	NM_016623	6291	5661	1.11	1.21E-07
49	WDR67***	NM_145647	1655	1423	1.16	1.67E-07
50	TMEM65.a	TMEM65.a	4117	3540	1.16	1.96E-07
51	GMNN	NM_015895	7458	5945	1.25	1.99E-07
52	PAGE4	NM_007003	6419	8065	0.80	2.00E-07
53	MYBPC1	NM_206821	8768	11120	0.79	2.61E-07
54	GPR137B	NM_003272	3997	3447	1.16	2.96E-07
55	ALAS1	NM_000688	5380	5035	1.07	3.55E-07
56	MSR1	NM_002445	3663	3025	1.21	3.65E-07
57	CDC2	NM_033379	1420	1130	1.26	3.90E-07
58	240093_x_at	240093_x_at	1789	1469	1.22	4.71E-07
59	IGFBP3	NM_000598	10673	9433	1.13	4.85E-07
60	RAP2B	NM_002886	3270	2922	1.12	5.00E-07
61	MGC14595.a***	MGC14595.a	2252	1995	1.13	5.46E-07
62	AZGP1	NM_001185	17252	20133	0.86	6.55E-07
63	NOX4	NM_016931	2321	1942	1.19	6.67E-07
64	STIP1	NM_006819	7630	7123	1.07	7.23E-07
65	PTPRN2	NM_130843	4471	5398	0.83	7.36E-07
66	CTNNB1	NM_001904	9989	9354	1.07	7.50E-07
67	C8orf76***	NM_032847	4088	3652	1.12	7.88E-07
68	YY1	NM_003403	9529	8635	1.10	8.08E-07

* The Gene ID is the accession number when available. Other Gene IDs can be found by searching the May 2004 assembly of the human genome at http://genome.ucsc.edu/cgi-bin/hgGateway.

** t-test

*** Genes mapped to 8q24

#### Systemic Progression Prediction Model Development and Testing on a Training set

A statistical model to predict systemic progression (with and without clinical variables) using a training set was developed using random forests [21] and logistic regression as described in Methods. The training data were analyzed by panel (cancer, custom and merged), by gene (the average expression for all gene-specific probes), and by individual probes. Table 4 lists the 15 genes and 2 individual probes selected for the final model.

**Table 4:** Final random forest 17 gene/probe model to predict prostate cancer systemic progression after a rising PSA following radical prostatectomy

				Mean DASL Expression Values		
Rank (t-test)	Symbol	Mean Gini Decrease*	p-value (t-test)	Systemic Progression	PSA Recurrence	Systemic:PSA Fold Change
1	RAD21**	2.15	8.57E-14	7587	6409	1.18
22	CDKN3	1.28	2.32E-09	1562	1229	1.27
15	CCNB1	1.25	3.65E-10	1871	1342	1.39
12	SEC14L1	1.14	8.20E-11	7248	6185	1.17
8	BUB1	1.06	2.07E-11	1257	957	1.31
55	ALAS1	1.04	3.55E-07	5380	5035	1.07
26	KIAA0196**	1.02	4.12E-09	5530	4945	1.12
3	TAF2**	1.02	6.99E-13	3144	2681	1.17
78	SFRP4	0.99	1.89E-06	15176	13059	1.16
64	STIP1	0.95	7.23E-07	7630	7123	1.07
25	CTHRC1	0.90	3.83E-09	3136	2480	1.26
4	SLC44A1	0.90	2.74E-12	4669	4022	1.17
5	IGFBP3	0.85	3.75E-12	4815	3782	1.27
307	EDG7	0.82	7.07E-03	5962	6757	0.88
48	FAM49B**	0.82	1.21E-07	6291	5661	1.11
19	C8ORF53**	0.97***	1.88E-09	7373	6444	1.14
275	CDK10	0.53***	4.12E-03	12254	12868	0.95

* Mean Gini Decrease for a variable is the average (over all random forest trees) decrease in node impurities from recursive partitioning splits on that variable. For classification, the node impurity is measured by the Gini index. The Gini index is the weighted average of the impurity in each branch, with impurity being the proportion of incorrectly classified samples in that branch. The larger the Gini decrease, the fewer the misclassification impurities.

** Genes mapped to 8q24

*** Single probes for C8orf53 and CDK10 were selected. The Mean Gini Decrease for these probes are derived from an independent random forest analysis of the all probes separately.

**Figure 2:**
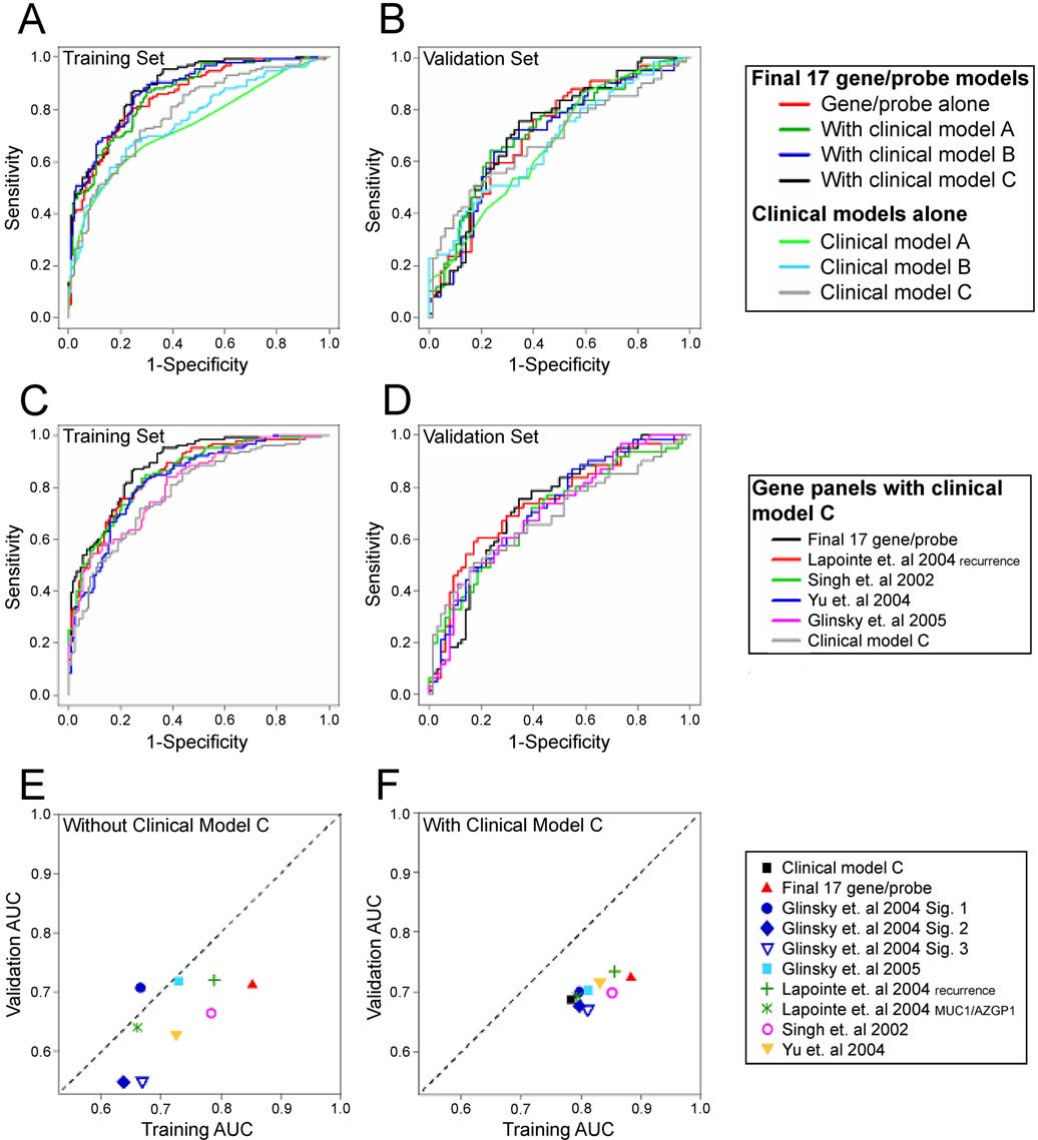
Areas under the curve (AUCs) for three clinical models, the final 17 gene/probe model and the combined clinical probe models. A. The training set AUCs for three clinical models, the final 17 gene/probe model and the combined clinical/17 gene/probe model. B. The validation set AUCs for three clinical models, the final 17 gene/probe model and the combined clinical/17 gene/probe model. C. The training set AUCs of 4 previously reported gene expression models of prostate cancer aggressiveness compared with the clinical model C alone and with the 17 gene/probe model. D. The validation set AUCs of 4 previously reported gene expression models of prostate cancer aggressiveness compared with the clinical model C alone and with the 17 gene/probe model. For an explanation of the clinical models see Table 6. (E and F) A comparison of the training and validation set AUCs for each of the model. E. AUCs of the each of the gene/probe models alone. F. AUCs of each of the gene/probe models with the inclusion of clinical model C.

Table 5 and Figure 2A summarize the areas under the curve (AUCs) for three clinical models, the final 17 gene/probe model and the combined clinical probe models. The variables in the clinical models (Table 6) were based on available clinical information. Clinical model A included revised Gleason score and pathologic stage (information available immediately after RRP). The addition of diagnostic PSA and age at surgery did not significantly add to the AUC and was left out of this model (data not shown). Clinical model B added age at surgery, preoperative PSA value, and any adjuvant or hormonal therapy within 90 days after RRP (information available after RRP but before PSA recurrence). Clinical model C added age at PSA recurrence, the second PSA level at time of PSA recurrence, and the PSA slope (information available at the time of PSA recurrence).

**Table 5:** Prediction of systemic progression–training set AUCs

	Probes alone	Clinical model*		
		A	B	C
Clinical model alone	**NA**	**0.736**	**0.757**	**0.783**
Final 17 gene/probe	**0.852**	**0.857**	**0.873**	**0.883**
Glinsky et al. 2004 Signature 1	**0.665**	**0.762**	**0.776**	**0.798**
Glinsky et al. 2004 Signature 2	**0.638**	**0.764**	**0.781**	**0.798**
Glinsky et al. 2004 Signature 3	**0.669**	**0.770**	**0.788**	**0.810**
Glinsky et al. 2005	**0.729**	**0.780**	**0.800**	**0.811**
Lapointe et al. 2004 Tumor Recurrence Sig.	**0.789**	**0.825**	**0.838**	**0.855**
Lapointe et al. 2004 (MUC1 and AZGP1)	**0.660**	**0.767**	**0.777**	**0.793**
Singh et al. 2002	**0.783**	**0.824**	**0.838**	**0.851**
Yu et al. 2004	**0.725**	**0.797**	**0.815**	**0.830**

* See Table 6 for clinical variables included in the clinical models

**Table 6:** Clinical variables included in clinical models

	Clinical model		
Clinical variable	A	B	C
Revised Gleason score	**X**	**X**	**X**
pStage	**X**	**X**	**X**
Age at surgery		**X**	**X**
Preoperative PSA		**X**	**X**
Hormone or radiation therapy after RRP		**X**	**X**
Age at PSA recurrence			**X**
Second PSA			**X**
PSA slope			**X**

Using the training set, clinical models A, B and C alone had AUCs of 0.74 (95% CI 0.68–0.80), 0.76 (95% CI 0.70–0.82) and 0.78 (95% CI 0.73–0.84), respectively. The 17 gene/probe model alone had an AUC of 0.85 (95% CI 0.81–0.90). When combined with the 17 gene/probe model, clinical models A, B, and C had AUCs of 0.86 (95% CI 0.81–0.90), 0.87 (95% CI 0.83–0.91) and 0.88 (95% CI 0.84–0.92), respectively. We also tested a 19 gene model that added TOP2A and survivin (BIRC5) to the17 gene/probe model. The addition of these two genes did not improve the prediction of systemic progression in the training set (data not shown).

The arrays were selected to include probe sets for several previously published prostate aggressiveness models [12]–[16]. Table 5 summarizes the AUCs for array expression results for these biomarker models. Figure 2C illustrates the AUCs for four of these models with the appropriate comparison with clinical model C and with the 17 gene/probe model. Each of these models generated AUCs that were smaller than the model we developed. However several of the models generated AUCs (e.g. the Lapointe et al. 2004 recurrence, Yu et al. 2004, and Singh et al. 2002 models) that were within or close to the 95% confidence limits of our AUC training set estimates.

#### Testing of Models on the Validation Set

We then applied the 17 gene/probe model and the other previously published models to the reserved 205 patient validation set (Figures 2B and 2D). Figure 2E compares the training set and validation set AUCs of the each of gene/probe models. With the exception of the Glinsky et al. 2004 Signature 1, all of the gene/probe models had significantly lower AUCs in the validation set compared to the training set. Figure 2F compares the training and validation set AUCs of each of the gene/probe models including clinical model C. While the 17 gene/probe model and three of the previously published models (the LaPointe et al. 2004 recurrence, Yu et al. 2004, and Glinsky et al. 2005 models) outperformed the clinical model alone, the AUCs were significantly lower in the validation set compared to the training set.

We also compared the models for their classification of patients into the known PSA recurrence control and SYS progression case groups. Table S4 summarizes the Cramér's V-statistic [27] of the various models, and includes a perfect predictor (“truth”) model for direct evaluation of the models. Briefly, the Cramér's V-statistic ranged from 0.38 to 0.70. The lowest Cramér's V-statistic was between the true state (perfect prediction) and the Glinsky et al. 2005 model with clinical data. The highest Cramér's V value was between our 17 gene/probe model and Singh et al. 2002 model, both with clinical data. Most of the models classified the same patients into the known groups (e.g. classifying a patient in the PSA control group as a PSA recurrence and a patient in the SYS case group as a systemic progression). They also tended to incorrectly classify the same patients (e.g classifying a patient in the PSA control group as a systemic progression and vice versa). The 17 gene/probe model correctly classified 5–15 more patients into their known category (PSA controls or SYS cases) compared to the other models (data not shown).

### Secondary Analyses

#### Exploratory Survival studies

As noted above, the 17 gene/probe model and the previously reported models each classified some of the SYS cases in the good outcome category (e.g. to be PSA recurrences, not systemic progressors) and some of the PSA controls in the poor outcome category (e.g. to go on to systemic progression). We were curious to know whether these apparently false classifications had any biologic or clinical relevance.

**Figure 3:**
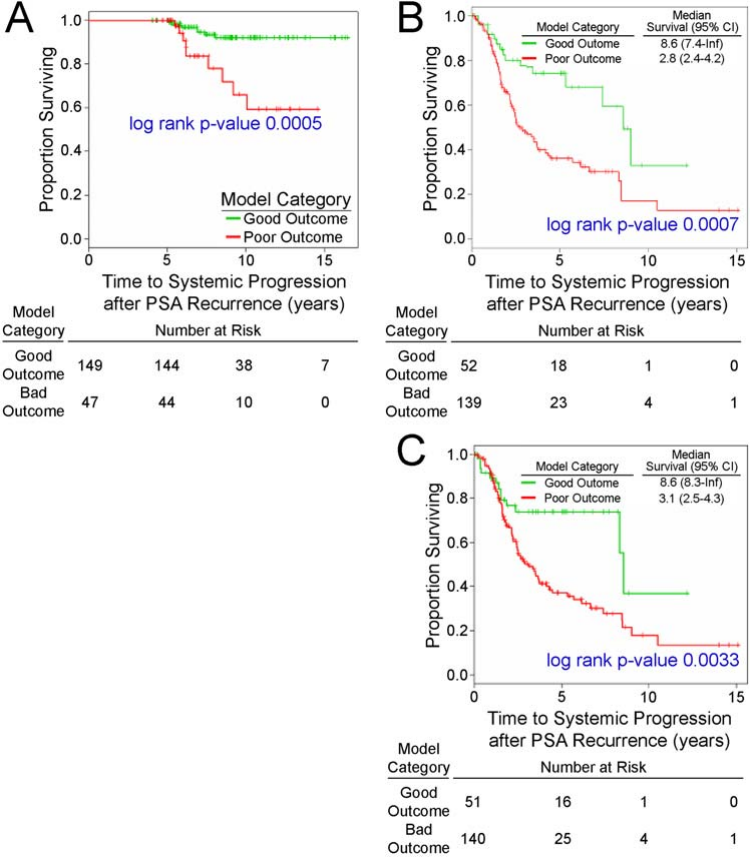
Systemic progression-free and overall prostate cancer-specific survival in the PSA control and SYS case groups. A) Systemic progression-free survival for the patients classified in the poor outcome category and for those in the good outcome category in the PSA control group–17 gene/probe model. B) Prostate cancer-specific overall survival for the patients classified in the poor outcome category and for those in the good outcome category in the SYS case group–17 gene/probe model. C) Prostate cancer-specific overall survival for patients classified in the poor outcome category and for those in the good outcome category in the SYS case group-Lapointe et al. 2004 recurrence model.

Seventeen men in the PSA control group (who had both array and clinical model C data) went on to have systemic progression beyond 5 years at the time of last follow-up. Of these 17 patients, 9 were predicted to have a poor outcome by the 17 gene/probe model. Of the 179 patients who did not have any systemic progression, 38 were classified in the poor outcome category by the model (p value = 0.0066, Fisher exact test). Figure 3A illustrates the systemic progression-free survival for the good and poor outcome groups in the PSA controls. PSA controls with a tumor classified as having a poor outcome had significantly increased risk for developing systemic progression beyond 5 years (log rank p-value = 0.00050) (HR = 4.7, 95% CI: 1.8–12.1).

Ninety-three men in the SYS case group (who also had array and clinical model C data) went on to prostate cancer death at the time of last follow-up. Of these 93 patients, 78 were predicted to have a poor outcome by the 17 gene/probe model. Of the 98 patients who did not suffer a prostate cancer death, 61 were classified in the poor outcome category by the model (p value = 0.0008, chi-square test). Figure 3B illustrates the prostate cancer-specific overall survival for the good and poor outcome groups in the SYS cases. SYS cases with a tumor classified as having a poor outcome had significantly increased risk for suffering a prostate cancer-specific death (HR = 2.5, 95% CI: 1.5–4.4). The median survival from first positive bone scan or CT was 2.8 years (95% CI: 2.4–4.2) in the group classified as having a poor outcome and 8.6 years (95% CI: 7.4–∞) in the group classified as having a good outcome (log rank p-value = 0.00068).

Similar associations were observed when three of the previously published models with high AUCs (the Lapointe et al. 2004 recurrence, Yu et al. 2004, and Glinsky et al. 2005 models) were evaluated. The following describes the results for the LaPointe et al. 2004 recurrence model (data for the other two models were similar and are not shown). Of the 98 patients who did not suffer a prostate cancer death, 60 were predicted to have a poor outcome by the Lapointe et al. 2004 recurrence model (p value = 0.0001, chi-square test). Figure 3C illustrates the prostate cancer-specific overall survival for the good and poor outcome groups in the SYS cases. SYS cases whose tumor classified as having a poor outcome had significantly increased hazard of suffering a prostate cancer-specific death (HR = 2.3, 95% CI: 1.3–4.2). The median survival from first positive bone scan or CT was 3.1 years (95% CI: 2.5–4.3) in the group classified as having a poor outcome and 8.6 years (95% CI: 8.3–∞) in the group classified as having a good outcome (log rank p-value = 0.0033).

#### Exploratory 8q24 Studies

Because of recent tumor chromosome dosage and germ line association studies, the custom array included 82 8q genes on the custom array. Fourteen 8q genes were within the top 68 genes based upon univariate analysis (Table 3). Compared to the proportion of 8q genes on both arrays the prevalence of 8q genes is non random (p = 0.003, Fisher exact test). Twelve additional 8q genes were within the top 126 genes. The prevalence of 26 8q genes in the top 126 is statistically significant (p = 1.56×10^−5^, Fisher exact test). Chromosome band 8q24.1 has the greatest overrepresentation of genes in the top 68 gene and 126 gene lists (11 genes, p = 6.35×10^−7^ and 19 genes, p = 9.34×10^−12^, Fisher exact test). Of the 17 genes/probes in our final model, five map to 8q24 (p = 0.0043, Fisher exact test)(see Table 4).

#### Exploratory ets Transcription Factor Studies

**Figure 4:**
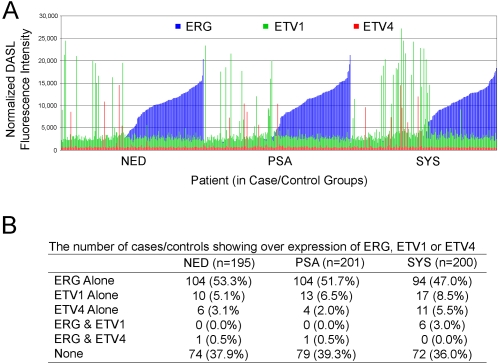
Expression results for ERG, ETV1 and ETV4 among the men with no evidence of disease progression (NED), PSA recurrence (PSA) and systemic progression (SYS). (A) Each overlapping set of three bars (blue, red and green) represent a different case or control. Thresholds for overexpression are ERG>3200, ETV1>6000 and ETV4>1400. (B) The numbers of cases showing overexpression of one or more of ERG, ETV1 and ETV4 are shown.

Alterations of several ets-family oncogenes are associated with the development of prostate cancer [28]–[30]. We included oligonucleotide probe sets for the three major members of the ets family involved in prostate cancer: ERG, ETV1, and ETV4, as well as their translocation partner TMPRSS2. Figure 4 summarizes the expression results for these genes for the SYS cases and the PSA and NED controls. Several observations can be made: 1) With only 8 exceptions ERG, ETV1 and ETV4 overexpression are mutually exclusive; i.e. the overexpression of each generally occurs in different tumors. 2) Different probe sets for ERG give nearly identical expression results (see Figure S4A). 3) The prevalence of ERG overexpression was 50.0%, 52.2% and 53.8% in the SYS cases, PSA controls and NED controls, respectively. There was no significant difference in the mean expression and the prevalence of ERG overexpression between the three cohorts (see Figure 4). 4) The prevalence of ETV1 overexpression was 11.5%, 6.5% and 5.1% in the SYS cases, PSA controls and NED controls, respectively (see Figure 4). The prevalence of ETV1 overexpression was significantly higher in SYS cases (p = 0.043, chi-square test). 5) The prevalance of ETV4 overexpression ranged from 2.5%–5.5% among the three groups and was not significantly different. 6) None of the genes were selected by the formal statistical modeling (see Table 4). In fact, the 17 gene/probe model predicted similar rates of progression in ERG+ and ERG-patients (data not shown).

#### Exploratory Pathway Analysis

We used the 461 genes from both cancer and custom panels that are potentially differentially expressed between SYS cases and PSA controls (p≤0.05) as the focus genes for Ingenuity Pathway Analysis (IPA, Ingenuity Systems Inc. Redwood City, CA). IPA identified 101 canonical pathways that are associated with the focus genes, 51 of which are overrepresented with p≤0.05 (see Table S5). However, because we measured a limited number of genes on both DASL panels, the p values from IPA analysis may not accurately quantify the degree of overrepresentation of focus genes in each pathway.

We then performed Gene Set Enrichment Analysis (GSEA) [31], on chromosome 8 genes grouped by map location. Genes mapped to 8q24.1 had a significant p value (p = 0.0002) with a FDR q value = 0.001 (see Table S6).

## Discussion

Patients with a rising PSA following definitive therapy comprise a heterogeneous cohort; a significant number develop metastasis, followed by hormone refractory prostate cancer. Of these, a substantial number, but not all, will die of the disease. PSA failure following RRP or radiation therapy is associated with a 15% to 25% five year prostate cancer death rate [32], [33]. Androgen deprivation has been increasingly used in all stages of prostate cancer to improve mortality rates [34], [35] or to facilitate prostate cytoreduction [36], [37]. Two recent studies described the natural history of progression after PSA elevation following RRP or radiation therapy [32], [38]. They identified PSA doubling time as a potential surrogate for prostate mortality. In three retrospective studies early androgen deprivation in patients with biochemical failure and short (<12 months) PSA doubling time after prostatectomy improved survival [39]–[41]. We hypothesized that additional biomarkers beyond PSA doubling time could help predict which men with a rising PSA post-RRP might suffer systemic progression. Such a panel could be incorporated into future prospective clinical trials in the setting of PSA progression.

Using an array methodology optimized for RNA from paraffin-embedded tissues and a rigorous statistical modeling algorithm, we developed a 17 gene/probe tissue gene expression model to predict the likelihood of systemic progression in men with a rising PSA post-RRP. In a training set the 17 gene/probe model was significantly better than the use of clinical variables alone. While accuracy decreased when the 17 gene/probe model was further tested with a reserved validation set, the performance of the 17 gene/probe model with clinical model C was better than the clinical model alone.

The reduction in AUC between the training and validation sets was in part due to the overfitting inherent in these types of analyses. Since we maximized the AUC on the training set, the validation set AUC would be predicted to be lower. Another cause of the reduction in AUC could be a relative lack of precision of the Illumina DASL technology. Except for the poor correlation of the DASL and RT-PCR measurements for genes with low ΔCt values, all of the intra-plate, inter-plate, intra-gene and inter-gene reproducibility analyses suggested that the DASL chemistry was very precise. We observed greater coefficients of variation in our replicate RT-PCR measurements than in the DASL measurements (data not shown).

Perhaps the best explanation for the validation set AUC reduction is that prostate cancer is genetically heterogeneous. This heterogeneity can result in a reduction in a validation set AUC even for relatively large datasets. This hypothesis predicts that several different models could be developed from the same dataset. One of the advantages of the Illumina DASL platform is its ability to analyze up to 1536 probes (or 512 genes if thee probes are selected per gene) on a single array. We included probes from eight previously reported gene expression panels associated with prostate cancer aggressiveness [12]–[16]. The models showed strong correlation with each other and generally predicted the same patients to be PSA recurrences or systemic progressors. A recent comparison of several breast cancer gene expression models by Fan et al. [42] also showed high correlation between models. The implication is that several independent biomarker panels can be developed to classify cancer patients with similar clinical or biologic endpoints.

After the formal analysis was completed we began secondary exploratory studies of the whole dataset. We hypothesized that systemic progression beyond 5 years in the PSA controls might be predicted by the models. When the 17 gene/probe model score (and three of the other models) predicted a poorer outcome, there was a high likelihood that a PSA control would have a positive bone scan or CT beyond 5 years. There was also evidence supporting a second hypothesis that prostate cancer-specific death in the SYS cases might be predicted by the models. When the 17 gene/probe model score (and three of the other models) predicted a poorer outcome, the median overall survival of a SYS case from a positive bone scan or CT was 2.8 years (compared to 8.6 years with a better model score). These secondary analyses imply that tissue expression biomarker panels may have utility for the stratification of patients for interventions at the time of PSA recurrence as well as for systemic progression. Importantly, the expression data was collected on primary tumor specimens resected several years before the occurrence of the clinical events.

Overrepresentation of 8q24 is associated with clinically aggressive prostate cancer (for example, references [43]–[45]). Furthermore, tumor overexpression of genes on chromosome 8 (and from 8q24) is also reproducibly associated with prostate cancer progression [46]–[49]. We recently mapped the region of 8q24 overrepresentation, and it involves a ∼5 Mb region surrounding c-Myc [50]. Our secondary analyses demonstrated that 8q24 genes were significantly overrepresented in the top 68 and 126 genes by t-test and in the final 17 gene/probe model. Each of the genes exhibited similar magnitudes of overexpression in the SYS cases suggesting an association with chromosomal dosage. A common germline polymorphism mapped near the c-Myc gene on 8q24, has recently been associated with prostate cancer development [51]. This finding has been replicated by at least five different groups, with the further suggestion that at least two germline haplotypes near c-Myc are associated with prostate cancer development [52]–[56]. It is not known if the men who inherit the at-risk haplotype(s) have a clinically more aggressive prostate cancer, a poorer prognosis, somatic (tumor) overrepresentation of 8q24, or overexpression of 8q24 genes.

Alterations of several ets-family oncogenes are associated with the development of prostate cancer [28]–[30]. Our panel(s) included probe sets for three members of the ets family involved in prostate cancer; ERG, ETV1, and ETV4, as well as their translocation partner, TMPRSS2. As a group, these genes are over-expressed in approximately 62% of prostate cancers. This overall prevalence was nearly identical in our three case and control groups. In addition, with the possible exception of ETV1, whose prevalence of overexpression was about 2-fold higher in the SYS cases, none of the genes seemed to be associated with systemic progression of prostate cancer. It has been recently reported that ERG fusion is associated with lethal prostate cancer in Scandinavian men treated with watchful waiting [30]. However, the prevalence of fusion (and presumably ERG overexpression) in that study was only 15%; far lower than in our dataset and other reports [28], [29]. These differences are likely a result of the types of prostate cancer (and clinical outcomes) diagnosed where PSA screening is common (North America) and uncommon (Scandinavia).

We conclude that the measurement of gene expression patterns may be useful for determining which men are likely to benefit from additional therapy following PSA recurrence. These measurements should be included in prospective evaluation of various therapeutic interventions when PSA rises following definitive treatment of prostate cancer.

## Supporting Information

Figure S1 Summary of the nested case-control study design.
(0.16 MB TIF)

Figure S2 Reproducibility of DASL assay and the effect of RNA quantity on the DASL assay. A) An example of DASL interplate reproducibility. B) Effect of reduced RNA quantity on the DASL assay.
(0.59 MB TIF)

Figure S3 Example results of the comparison of quantitative RT-PCR and DASL data. ERG-Cancer Panel ver1 (A, R2 = 0.94), ERG-Custom Panel (B, R2 = 0.94), PAGE4 (C, R2 = 0.89), MUC1 (D, R2 = 0.82) and FAM13C1 (E, R2 = 0.75). (F) Summary of quantitative RT-PCR and DASL data comparisons.
(0.48 MB TIF)

Figure S4 Comparison of genes having multiple probe sets on the Cancer Panel v1 and/or the Custom panel. A) Comparison of three probe sets (Cancer Panel ERG, Custom panel ERG and Custom panel ERG splice variant) for ERG. B) Comparison of two probe sets (Custom Panel SRD5A2 and Custom panel terparbo) for SRD5A2/terparbo.
(2.13 MB TIF)

Results S1 The Supplemental Results describe the replicate analysis results, RT-PCR comparisons and inter- and intra-panel gene expression comparisons.
(0.05 MB DOC)

Table S1 The List of Genes Included on the Commercially Available Illumina DASL Cancer Panel v1. Prostate cancer relevant genes are indicated (for selection criteria see footnotes following Table S2).
(0.11 MB XLS)

Table S2 Genes Relevant to Prostate Cancer Progression Included on an Illumina DASL Custom Array (for selection criteria see footnotes below).
(0.16 MB XLS)

Table S3 Genes from Commercially Available Illumina DASLTM Cancer Panel and Illumina DASL Custom Array Ranked by Increasing P-Value.
(0.21 MB XLS)

Table S4 Cramér's V-statistic for selection between PSA recurrence and systemic progression. All samples are included (both training and validation sets). All models were augmented with clinical information.
(0.02 MB PDF)

Table S5 The Top 51 pathways associated with systemic progression by Ingenuity Pathway Analysis.
(0.03 MB DOC)

Table S6 Association of genes on chromosome 8 with systemic progression using Gene Set Enrichment Analysis (GSEA).
(0.02 MB PDF)

## References

[1] Jemal A, Murray T, Ward E, Samuels A, Tiwari RC (2005). Cancer statistics.. CA Cancer J Clin.

[2] D'Amico AV, Moul J, Carroll PR, Sun L, Lubeck D (2003). Cancer-specific mortality after surgery or radiation for patients with clinically localized prostate cancer managed during the prostate-specific antigen era.. J Clin Oncol.

[3] Amling CL, Blute ML, Bergstralh EJ, Seay TM, Slezak J (2000). Long-term hazard of progression after radical prostatectomy for clinically localized prostate cancer: continued risk of biochemical failure after 5 years.. J Urol.

[4] Shipley WU, Thames HD, Sandler HM, Hanks GE, Zietman AL (1999). Radiation therapy for clinically localized prostate cancer: a multi-institutional pooled analysis.. JAMA.

[5] Patel DA, Presti JC, McNeal JE, Gill H, Brooks JD (2005). Preoperative PSA velocity is an independent prognostic factor for relapse after radical prostatectomy.. J Clin Oncol.

[6] Moul JW (2000). Prostate specific antigen only progression of prostate cancer.. J Urol.

[7] Dhanasekaran SM, Barrette TR, Ghosh D, Shah R, Varambally S (2001). Delineation of prognostic biomarkers in prostate cancer.. Nature.

[8] Luo J, Duggan DJ, Chen Y, Sauvageot J, Ewing CM (2001). Human prostate cancer and benign prostatic hyperplasia: molecular dissection by gene expression profiling.. Cancer Res.

[9] Magee JA, Araki T, Patil S, Ehrig T, True L (2001). Expression profiling reveals hepsin overexpression in prostate cancer.. Cancer Res.

[10] Welsh JB, Sapinoso LM, Su AI, Kern SG, Wang-Rodriguez J (2001). Analysis of gene expression identifies candidate markers and pharmacological targets in prostate cancer.. Cancer Res.

[11] LaTulippe E, Satagopan J, Smith A, Scher H, Scardino P (2002). Comprehensive gene expression analysis of prostate cancer reveals distinct transcriptional programs associated with metastatic disease.. Cancer Res.

[12] Singh D, Febbo PG, Ross K, Jackson DG, Manola J (2002). Gene expression correlates of clinical prostate cancer behavior.. Cancer Cell.

[13] Glinsky GV, Glinskii AB, Stephenson AJ, Hoffman RM, Gerald WL (2004). Gene expression profiling predicts clinical outcome of prostate cancer.. J Clin Invest.

[14] Lapointe J, Li C, Higgins JP, van de Rijn M, Bair E (2004). Gene expression profiling identifies clinically relevant subtypes of prostate cancer.. Proc Natl Acad Sci U S A.

[15] Yu YP, Landsittel D, Jing L, Nelson J, Ren B (2004). Gene expression alterations in prostate cancer predicting tumor aggression and preceding development of malignancy.. J Clin Oncol.

[16] Glinsky GV, Berezovska O, Glinskii AB (2005). Microarray analysis identifies a death-from-cancer signature predicting therapy failure in patients with multiple types of cancer.. J Clin Invest.

[17] Bibikova M, Talantov D, Chudin E, Yeakley JM, Chen J (2004). Quantitative gene expression profiling in formalin-fixed, paraffin-embedded tissues using universal bead arrays.. Am J Pathol.

[18] Bibikova M, Yeakley JM, Chudin E, Chen J, Wickham E (2004). Gene expression profiles in formalin-fixed, paraffin-embedded tissues obtained with a novel assay for microarray analysis.. Clin Chem.

[19] Bibikova M, Chudin E, Arsanjani A, Zhou L, Garcia EW (2007). Expression signatures that correlated with Gleason score and relapse in prostate cancer.. Genomics.

[20] Kube DM, Savci-Heijink CD, Lamblin AF, Kosari F, Vasmatzis G (2007). Optimization of laser capture microdissection and RNA amplification for gene expression profiling of prostate cancer.. BMC Mol Biol.

[21] Rhodes DR, Yu J, Shanker K, Deshpande N, Varambally R (2004). ONCOMINE: A Cancer Microarray Database and Data-Mining Platform.. Neoplasia.

[22] Rhodes DR, Yu J, Shanker K, Deshpande N, Varambally R (2004). Large-Scale Meta-Analysis of Cancer Microarray Data Identifies Common Transcriptional Profiles of Neoplastic Transformation and Progression.. Proc Natl Acad Sci U S A.

[23] Tollefson MK, Slezak JM, Leibovich BC, Zincke H, Blute ML (2007). Stratification of patient risk based on prostate-specific antigen doubling time after radical retropubic prostatectomy.. Mayo Clin Proc.

[24] Bergstralh EJ, Kosanke JL, Jacobsen SJ (1995). Software for optimal matching in observational studies.. Epidemiology.

[25] Ballman KV, Grill DE, Oberg AL, Therneau TM (2004). Faster cyclic loess: normalizing RNA arrays via linear models.. Bioinformatics.

[26] Breiman L (2001). Random Forests.. Machine Learning.

[27] Cramér H (1999). Mathematical Methods of Statistics..

[28] Tomlins SA, Rhodes DR, Perner S, Dhanasekaran SM, Mehra R (2005). Recurrent fusion of TMPRSS2 and ETS transcription factor genes in prostate cancer.. Science.

[29] Tomlins SA, Mehra R, Rhodes DR, Smith LR, Roulston D (2006). TMPRSS2:ETV4 gene fusions define a third molecular subtype of prostate cancer.. Cancer Res.

[30] Demichelis F, Fall K, Perner S, Andren O, Schmidt F, Setlur SR (2007). TMPRSS2:ERG gene fusion associated with lethal prostate cancer in a watchful waiting cohort.. Oncogene.

[31] Subramanian A, Tamayo P, Mootha VK, Mukherjee S, Ebert BL (2005). Gene set enrichment analysis: a knowledge-based approach for interpreting genome-wide expression profiles.. Proc Natl Acad Sci U S A.

[32] Pound CR, Partin AW, Eisenberger MA, Chan DW, Pearson JD (1999). Natural history of progression after PSA elevation following radical prostatectomy.. JAMA.

[33] Sandler HM, Dunn RL, McLaughlin PW, Hayman JA, Sullivan MA (2000). Overall survival after prostate-specific-antigen-detected recurrence following conformal radiation therapy.. Int J Rad Oncol Biol Phys.

[34] Pilepich MV, Winter K, John MJ, Mesic JB, Sause W (2001). Phase III radiation therapy oncology group (RTOG) trial 86-10 of androgen deprivation adjuvant to definitive radiotherapy in locally advanced carcinoma of the prostate.. Int J Rad Oncol Biol Phys.

[35] Lawton CA, Winter K, Murray K, Machtay M, Mesic JB (2001). Updated results of the phase III Radiation Therapy Oncology Group (RTOG) trial 85-31 evaluating the potential benefit of androgen suppression following standard radiation therapy for unfavorable prognosis carcinoma of the prostate.. Int J Rad Oncol Biol Phys.

[36] Zelefsky MJ, Leibel SA, Burman CM, Kutcher GJ, Harrison A (1994). Neoadjuvant hormonal therapy improves the therapeutic ratio in patients with bulky prostatic cancer treated with three-dimensional conformal radiation therapy.. Int J Radiat Oncol Biol Phys.

[37] Gleave ME, Goldenberg SL, Chin JL, Warner J, Saad F (2001). Randomized comparative study of 3 versus 8-month neoadjuvant hormonal therapy before radical prostatectomy: biochemical and pathological effects.. J Urol.

[38] D'Amico AV, Cote K, Loffredo M, Renshaw AA, Schultz D (2002). Determinants of prostate cancer-specific survival after radiation therapy for patients with clinically localized prostate cancer.. J Clin Oncol.

[39] Pinover WH, Horwitz EM, Hanlon AL, Uzzo RG, Hanks GE (2003). Validation of a treatment policy for patients with prostate specific antigen failure after three-dimensional conformal prostate radiation therapy.. Cancer..

[40] Kestin LL, Vicini FA, Martinez AA (2004). Potential survival advantage with early androgen deprivation for biochemical failure after external beam radiotherapy: the importance of accurately defining biochemical disease status.. Int J Rad Oncol Biol Phys.

[41] Moul JW, Wu H, Sun L, McLeod DG, Amling C (2004). Early versus delayed hormonal therapy for prostate specific antigen only recurrence of prostate cancer after radical prostatectomy.. J Urol.

[42] Fan C, Oh DS, Wessels L, Weigelt B, Nuyten DS (2006). Concordance among gene-expression-based predictors for breast cancer.. N Engl J Med.

[43] Sato K, Qian J, Slezak JM, Lieber MM, Bostwick DG (1999). Clinical significance of alterations of chromosome 8 in high-grade, advanced, nonmetastatic prostate carcinoma.. J Natl Cancer Inst.

[44] Saramaki O, Willi N, Bratt O, Gasser TC, Koivisto P (2001). Amplification of EIF3S3 gene is associated with advanced stage in prostate cancer.. Am J Pathol.

[45] Tsuchiya N, Slezak JM, Lieber MM, Bergstralh EJ, Jenkins RB (2002). Clinical significance of alterations of chromosome 8 detected by fluorescence *in situ* hybridization analysis in pathologic organ-confined prostate cancer.. Genes Chromosomes Cancer.

[46] Porkka KP, Tammela TL, Vessella RL, Visakorpi T (2007). RAD21 and KIAA0196 at 8q24 are amplified and overexpressed in prostate cancer.. Genes Chromosomes Cancer..

[47] Savinainen KJ, Linja MJ, Saramaki OR, Tammela TL, Chang GT (2004). Expression and copy number analysis of TRPS1, EIF3S3 and MYC genes in breast and prostate cancer.. Br J Cancer.

[48] Savinainen KJ, Helenius MA, Lehtonen HJ, Visakorpi T (2006). Overexpression of EIF3S3 promotes cancer cell growth.. Prostate.

[49] Tomlins SA, Mehra R, Rhodes DR, Cao X, Wang L (2007). Integrative molecular concept modeling of prostate cancer progression.. Nat Genet.

[50] Tsuchiya N, Kondo Y, Takahashi A, Pawar H, Qian J (2002). Mapping and gene expression profile of the minimally overrepresented 8q24 region in prostate cancer.. Am J Pathol.

[51] Amundadottir LT, Sulem P, Gudmundsson J, Helgason A, Baker A (2006). A common variant associated with prostate cancer in European and African populations.. Nat Genet.

[52] Haiman CA, Patterson N, Freedman ML, Myers SR, Pike MC (2007). Multiple regions within 8q24 independently affect risk for prostate cancer.. Nat Genet.

[53] Schumacher FR, Feigelson HS, Cox DG, Haiman CA, Albanes D (2007). A Common 8q24 Variant in Prostate and Breast Cancer from a Large Nested Case-Control Study.. Cancer Res.

[54] Severi G, Hayes VM, Padilla EJ, English DR, Southey MC (2007). The common variant rs1447295 on chromosome 8q24 and prostate cancer risk: results from an Australian population-based case-control study.. Cancer Epidemiol Biomarkers Prev.

[55] Wang L, McDonnell SK, Slusser JP, Hebbring SJ, Cunningham JM (2007). Two common chromosome 8q24 variants are associated with increased risk for prostate cancer.. Cancer Res.

[56] Yeager M, Orr N, Hayes RB, Jacobs KB, Kraft P (2007). Genome-wide association study of prostate cancer identifies a second risk locus at 8q24.. Nat Genet.

