# Supplementary figures and images for: A Tissue Biomarker Panel Predicting Systemic Progression after PSA Recurrence Post-Definitive Prostate Cancer Therapy
Source: PLoS One. 2008 May 28;3(5):e2318. doi: 10.1371/journal.pone.0002318 (PMC2565588; doi:10.1371/journal.pone.0002318)

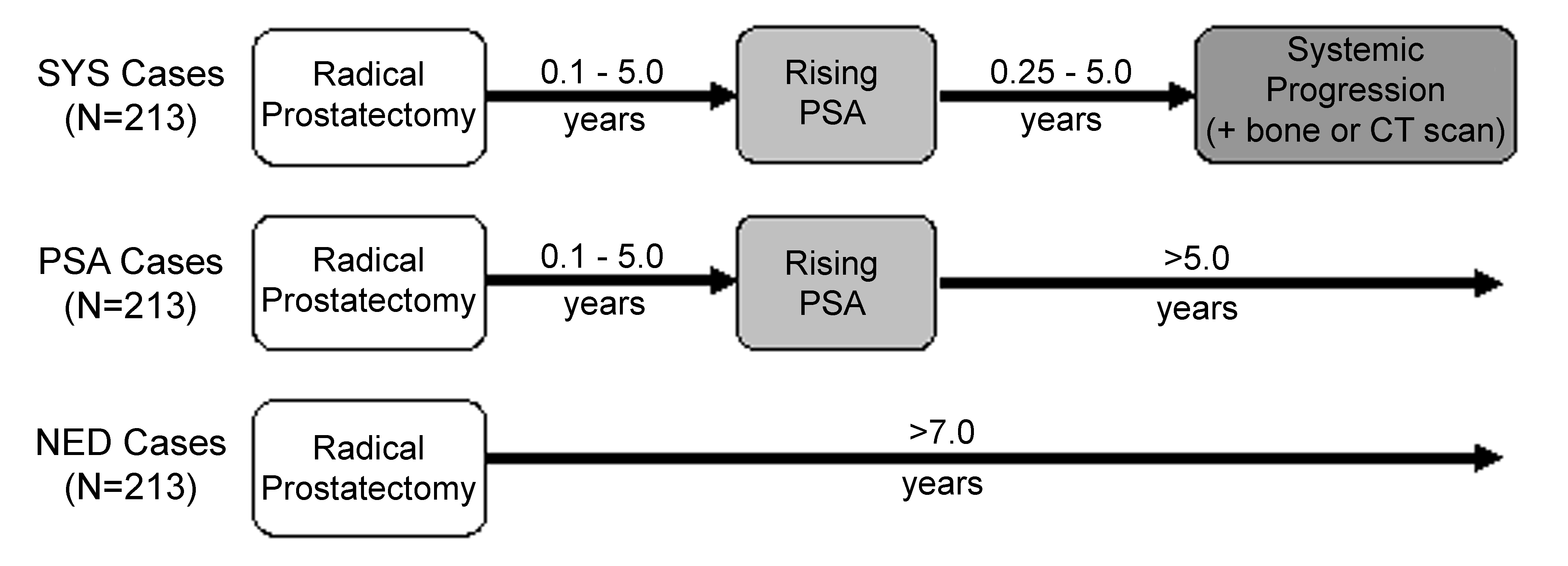

Supplement: Figure S1 — Summary of the nested case-control study design. (0.16 MB TIF) [file pone.0002318.s001.tif]

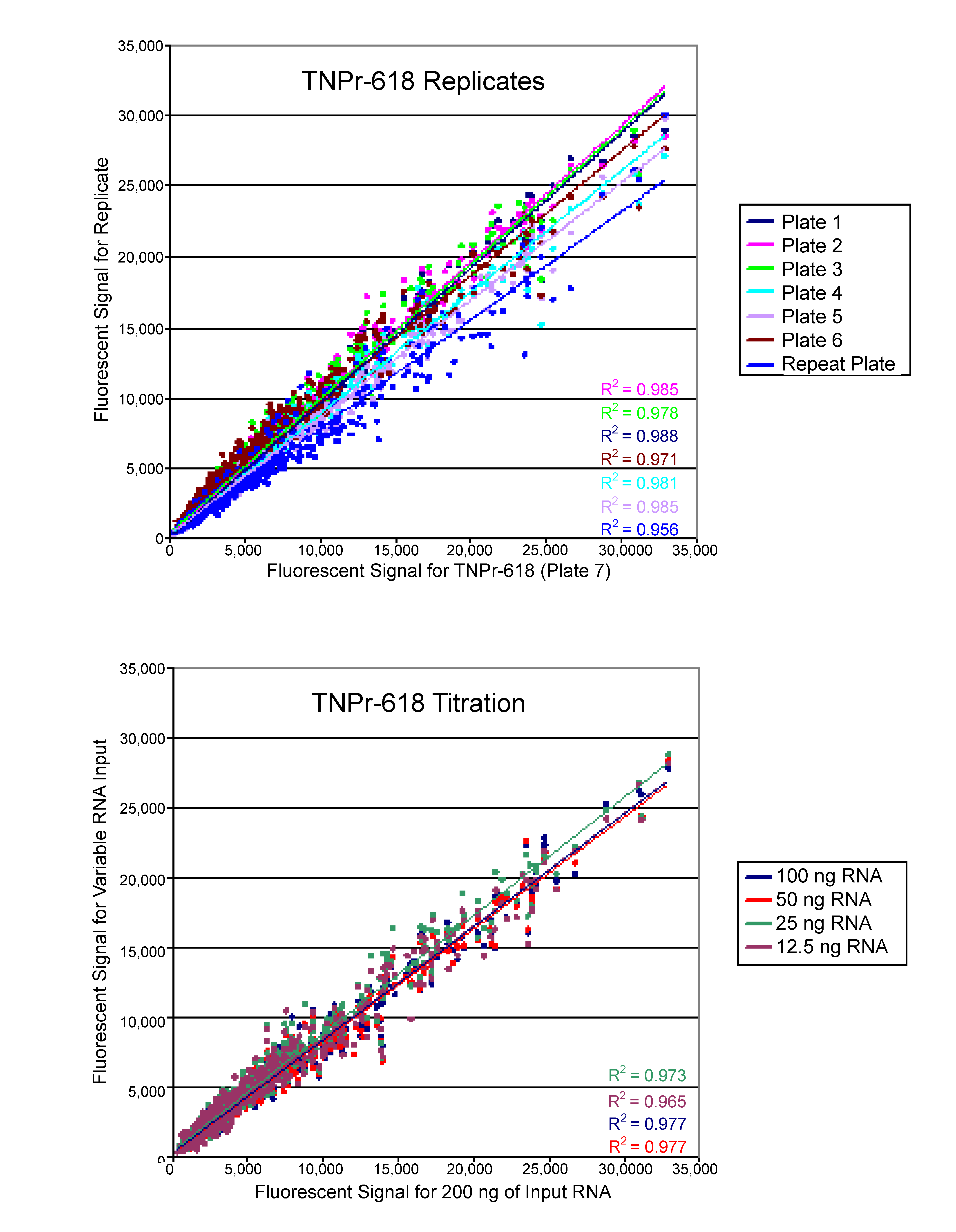

Supplement: Figure S2 — Reproducibility of DASL assay and the effect of RNA quantity on the DASL assay. A) An example of DASL interplate reproducibility. B) Effect of reduced RNA quantity on the DASL assay. (0.59 MB TIF) [file pone.0002318.s002.tif]

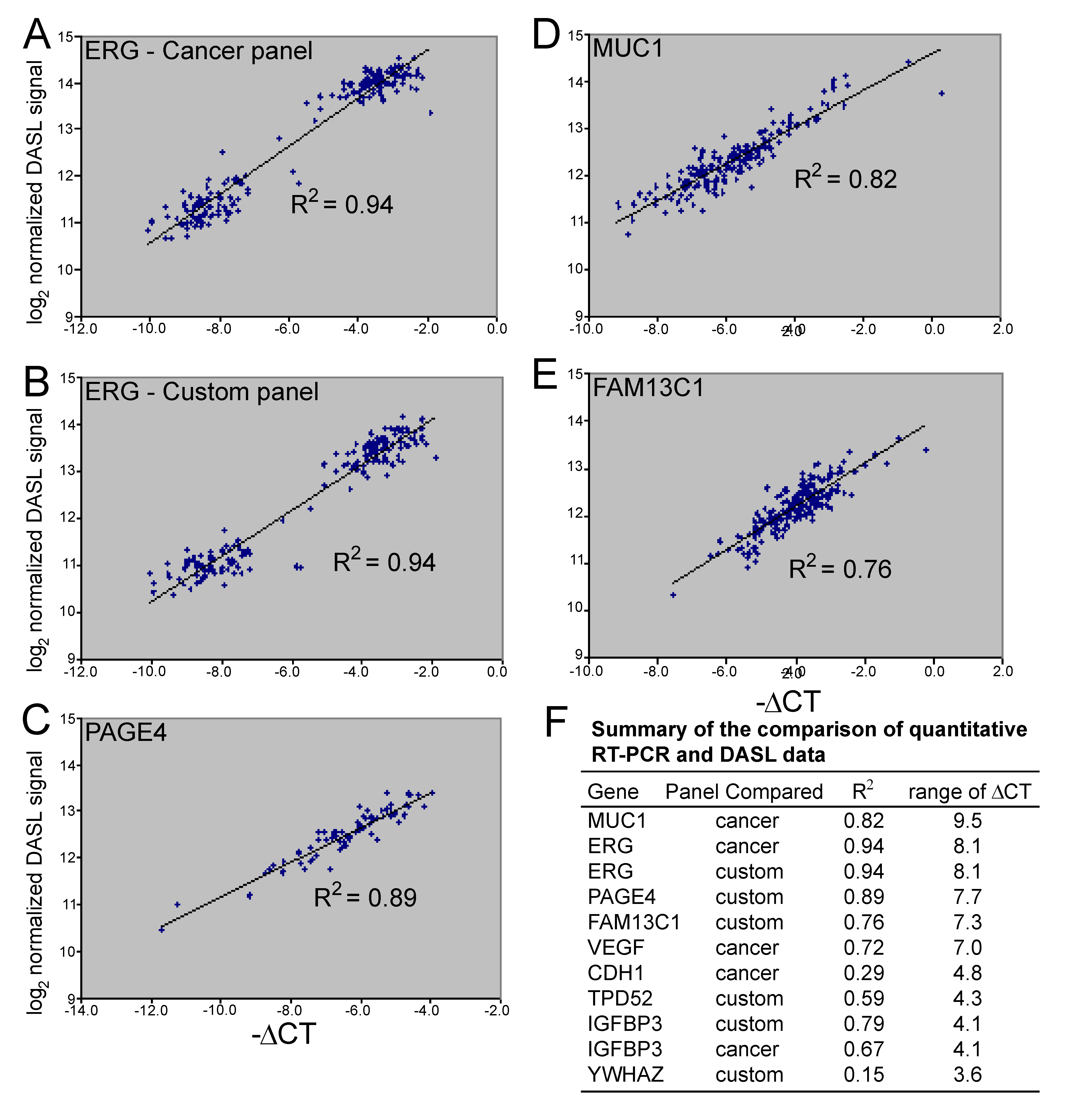

Supplement: Figure S3 — Example results of the comparison of quantitative RT-PCR and DASL data. ERG-Cancer Panel ver1 (A, R2 = 0.94), ERG-Custom Panel (B, R2 = 0.94), PAGE4 (C, R2 = 0.89), MUC1 (D, R2 = 0.82) and FAM13C1 (E, R2 = 0.75). (F) Summary of quantitative RT-PCR and DASL data comparisons. (0.48 MB TIF) [file pone.0002318.s003.tif]

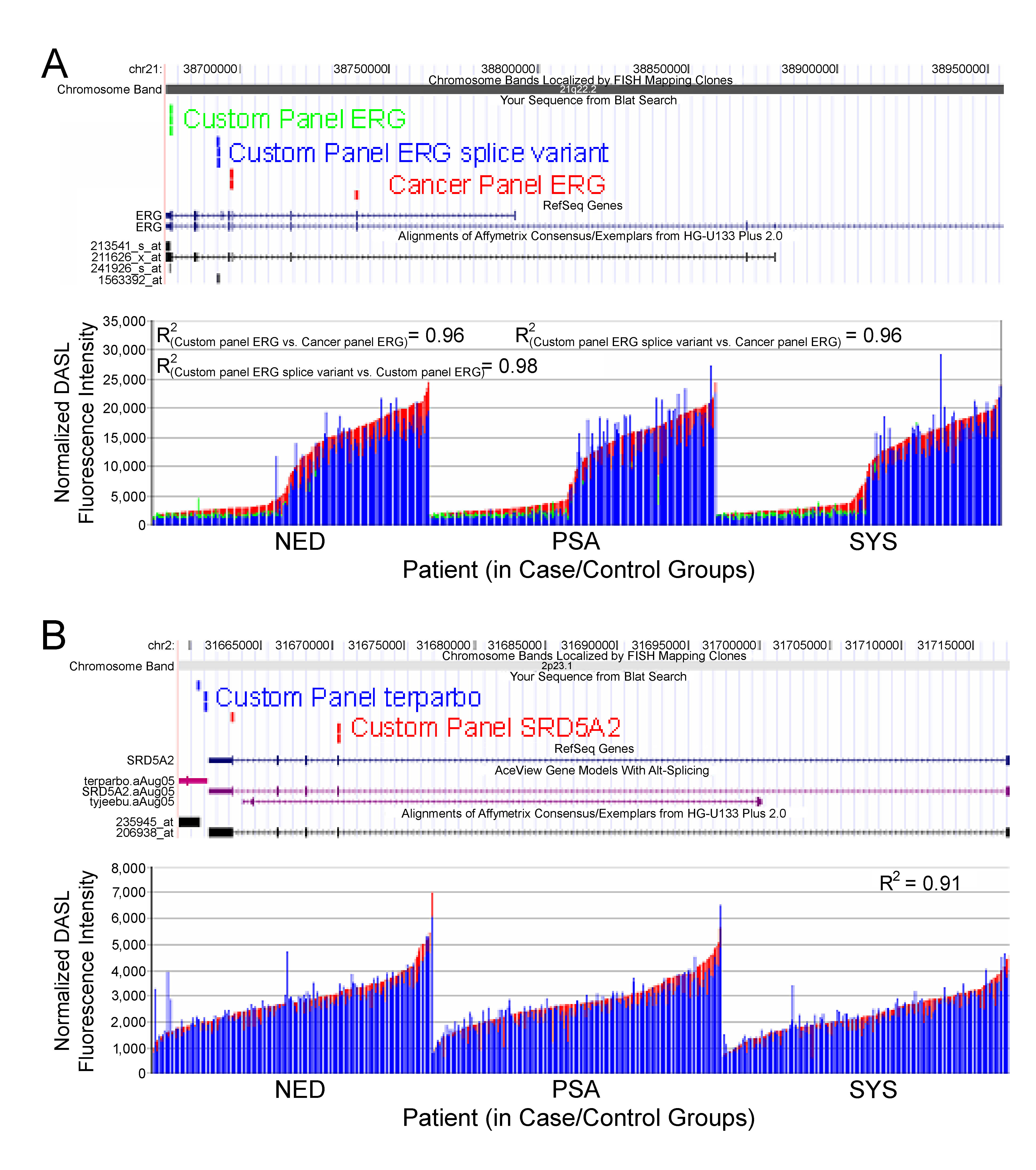

Supplement: Figure S4 — Comparison of genes having multiple probe sets on the Cancer Panel v1 and/or the Custom panel. A) Comparison of three probe sets (Cancer Panel ERG, Custom panel ERG and Custom panel ERG splice variant) for ERG. B) Comparison of two probe sets (Custom Panel SRD5A2 and Custom panel terparbo) for SRD5A2/terparbo. (2.13 MB TIF) [file pone.0002318.s004.tif]
