# Supplementary material for: A Tissue Biomarker Panel Predicting Systemic Progression after PSA Recurrence Post-Definitive Prostate Cancer Therapy
Source: PLoS One. 2008 May 28;3(5):e2318. doi: 10.1371/journal.pone.0002318 (PMC2565588; doi:10.1371/journal.pone.0002318)
Supplement: Results S1 — The Supplemental Results describe the replicate analysis results, RT-PCR comparisons and inter- and intra-panel gene expression comparisons. (0.05 MB DOC) [file pone.0002318.s005.doc]

**Results S1: Replicate analysis results, RT-PCR comparisons and Inter- and Intra-panel gene expression comparisons**

*Replicate analyses:* The study design included several intra- and inter-run array replicates. To determine inter-run array variability, two specimens were run on each of 8 Cancer Panel v1 array runs. The median (range) inter-run correlation coefficients (r^2^) comparing these two specimen replicates were 0.94 (0.89 -0.95) and 0.98 (0.90 – 0.98), respectively. The same two specimens were run on each of 8 custom prostate cancer panel array runs. The median (range) inter-run correlation coefficients (r^2^) comparing these specimen replicates were 0.97 (0.95 – 0.98) and 0.98 (0.96 – 0.99), respectively. Figure S2A summarizes the inter-run replicates for one of the specimens on the custom panel. Twelve specimens were evaluated as intra-run array replicates. The median (range) intra-run r^2^ values comparing these paired specimens on the Cancer Panel v1 was 0.98 (0.93 – 0.99). The median (range) intra-run r^2^ values comparing these paired specimens on the custom panel was 0.98 (0.88 – 0.99). Two specimens were serially diluted and the expression results of the diluted RNA specimens compared to that of the standard 200ng of the parental RNA specimen. The r^2^ for RNA specimens of 25, 50, and 100 ng ranged from 0.98-0.99 (Figure S2B) with slopes near 1.0.

*Comparison with RT-PCR*: RT-PCR analyses were performed for 9 genes (*CDH1, VEGF, MUC1, IGFBP3, ERG, TPD52, YWHAZ, FAM13C1*, and *PAGE4*) on 210 samples. Example results are illustrated in Figure S3. Comparison of the quantitative RT-PCR and the DASL results gave r^2^ values of 0.72 - 0.94 for genes with dynamic range of at least 7 ΔCTs. Genes with a smaller dynamic range of ΔCT gave r^2^ values of 0.15-0.79 (Figure S3). Thus, both the DASL and RT-PCR measurements appear to be highly correlated with each other when there is a broad range of RNA expression values.

*Inter- and Intra-Panel Gene Expression Comparisons:* By design several genes were evaluated twice on the custom and/or cancer panels. As an example of a specific inter-panel gene expression comparison, probe sets for *ERG* were present on both the custom (two 3 probe sets) and cancer (one 3 probe set) panels. The r^2^ comparing the 2 custom probe sets with the commercial probe set for all 596 patients was 0.96 in both cases (Figure S4A). As an example of a specific intra-custom panel gene expression comparison are the probe sets for *SRD5A2* and *terparbo*. *terparbo* is a “novel” gene which is likely a variant of the *SRD5A2* transcript (UCSC browser, http://genome.ucsc.edu). The r^2^ comparing the two custom probe sets for *SRD5A2* and terparbo was 0.91 (Figure S4B).
