# Supplementary material for: A Tissue Biomarker Panel Predicting Systemic Progression after PSA Recurrence Post-Definitive Prostate Cancer Therapy
Source: PLoS One. 2008 May 28;3(5):e2318. doi: 10.1371/journal.pone.0002318 (PMC2565588; doi:10.1371/journal.pone.0002318)
Supplement: Table S4 — Cramér's V-statistic for selection between PSA recurrence and systemic progression. All samples are included (both training and validation sets). All models were augmented with clinical information. (0.02 MB PDF) [file pone.0002318.s009.pdf]

**Table S4: Cramér's V-statistic for selection between PSA recurrence and systemic progression. All samples are included (both training and validation sets). All models were augmented with clinical information.**

| | Model 2 | | | | |
|---|---|---|---|---|---|
| Model 1 | Final 17 gene/probe | Glinsky et al. 2005 | Lapointe et al. 2004 recurrence signature | Singh et al. 2002 | Yu et al. 2004 |
| Truth (Known Cohort) | 0.50 | 0.38 | 0.47 | 0.43 | 0.44 |
| Final 17 gene/probe | | 0.65 | 0.64 | 0.70 | 0.64 |
| Glinsky et al. 2005 | | | 0.61 | 0.66 | 0.67 |
| Lapointe et al. 2004 recurrence signature | | | | 0.69 | 0.68 |
| Singh et al. 2002 | | | | | 0.66 |
