# Supplementary material for: A Tissue Biomarker Panel Predicting Systemic Progression after PSA Recurrence Post-Definitive Prostate Cancer Therapy
Source: PLoS One. 2008 May 28;3(5):e2318. doi: 10.1371/journal.pone.0002318 (PMC2565588; doi:10.1371/journal.pone.0002318)
Supplement: Table S5 — The Top 51 pathways associated with systemic progression by Ingenuity Pathway Analysis. (0.03 MB DOC) [file pone.0002318.s010.doc]

**Table S5: The Top 51 pathways associated with systemic progression by Ingenuity Pathway Analysis.**

Pathway p-Value Number of Number of

genes from genes from data pathway

PI3K/AKT Signaling 1.26E-16 26 144

Aryl Hydrocarbon Receptor Signaling 1.26E-14 24 147

p53 Signaling 1.00E-13 19 87

Apoptosis Signaling 6.31E-13 20 111

TGF-β Signaling 1.26E-12 19 106

PTEN Signaling 3.98E-12 19 109

IGF-1 Signaling 1.58E-10 16 90

IL-6 Signaling 2.69E-10 16 93

B Cell Receptor Signaling 1.32E-09 19 148

Cell Cycle: G1/S Checkpoint Regulation 2.88E-09 12 57

Death Receptor Signaling 1.02E-08 12 62

Cell Cycle: G2/M DNA Damage

Checkpoint Regulation 1.05E-08 10 42

Integrin Signaling 1.78E-08 21 211

Ephrin Receptor Signaling 1.95E-08 20 207

Axonal Guidance Signaling 2.57E-08 29 407

ERK/MAPK Signaling 3.89E-08 20 202

SAPK/JNK Signaling 3.89E-08 16 130

PPAR Signaling 7.41E-08 13 93

T Cell Receptor Signaling 1.07E-07 14 104

NF-κB Signaling 1.15E-07 16 139

PPARα/RXRα Activation 1.55E-07 16 145

Wnt/β-catenin Signaling 1.70E-07 17 162

IL-2 Signaling 2.63E-07 10 53

PDGF Signaling 1.00E-06 11 75

FGF Signaling 3.63E-06 11 84

Actin Cytoskeleton Signaling 3.80E-06 19 254

Neuregulin Signaling 5.13E-06 11 89

Huntington's Disease Signaling 1.20E-05 17 229

IL-10 Signaling 2.57E-05 9 70

Toll-like Receptor Signaling 2.75E-05 8 53

VEGF Signaling 2.95E-05 10 90

Neurotrophin/TRK Signaling 3.63E-05 9 72

GM-CSF Signaling 6.92E-05 8 62

EGF Signaling 9.55E-05 7 47

Insulin Receptor Signaling 1.55E-04 11 125

Chemokine Signaling 3.39E-04 8 75

p38 MAPK Signaling 3.55E-04 9 95

JAK/Stat Signaling 3.72E-04 7 59

Fc Epsilon RI Signaling 4.79E-04 9 100

G-Protein Coupled Receptor Signaling 7.41E-04 13 200

NRF2-mediated Oxidative Stress Response 1.07E-03 10 142

Hypoxia Signaling in the

Cardiovascular System 4.57E-03 6 68

Nicotinate and Nicotinamide Metabolism 4.79E-03 8 129

Sonic Hedgehog Signaling 4.79E-03 4 31

Leukocyte Extravasation Signaling 1.12E-02 10 186

Estrogen Receptor Signaling 1.29E-02 7 113

Natural Killer Cell Signaling 1.41E-02 7 110

IL-4 Signaling 1.51E-02 5 64

Inositol Phosphate Metabolism 1.58E-02 9 178

Xenobiotic Metabolism Signaling 1.62E-02 11 228

Amyotrophic Lateral Sclerosis Signaling 3.23E-02 6 103
