# Supplementary material for: A Tissue Biomarker Panel Predicting Systemic Progression after PSA Recurrence Post-Definitive Prostate Cancer Therapy
Source: PLoS One. 2008 May 28;3(5):e2318. doi: 10.1371/journal.pone.0002318 (PMC2565588; doi:10.1371/journal.pone.0002318)
Supplement: Table S6 — Association of genes on chromosome 8 with systemic progression using Gene Set Enrichment Analysis (GSEA). (0.02 MB PDF) [file pone.0002318.s011.pdf]

**Table S6: Association of genes on chromosome 8 with systemic progression using Gene Set Enrichment Analysis (GSEA).**

| Location | Number of genes on panels | Enrichment score (ES) | Normalized ES | Nominal p-value | False discovery rate q-value |
|---|---|---|---|---|---|
| 8p | 15 | -0.32 | -0.85 | 0.65 | 1.00 |
| 8p23 | 2 | 0.77 | 1.15 | 0.30 | 0.36 |
| 8p22 | 3 | 0.59 | 0.99 | 0.53 | 0.55 |
| 8p21 | 7 | -0.67 | -1.43 | 0.10 | 0.27 |
| 8p12 | 2 | -0.81 | -1.22 | 0.21 | 0.51 |
| 8p11 | 1 | 0.73 | 0.95 | 0.59 | 0.54 |
| 8q | 75 | 0.68 | 1.92 | 3.94E-03 | 2.09E-03 |
| 8q11 | 1 | -0.51 | -0.69 | 0.98 | 1.00 |
| 8q12 | 3 | -0.38 | -0.67 | 0.87 | 0.87 |
| 8q13 | 1 | 0.92 | 1.21 | 0.18 | 0.37 |
| 8q21 | 5 | 0.60 | 1.20 | 0.27 | 0.33 |
| 8q22 | 4 | 0.90 | 1.57 | 8.72E-03 | 4.63E-02 |
| 8q23 | 4 | 0.76 | 1.34 | 0.13 | 0.20 |
| 8q24 | 57 | 0.69 | 1.90 | 2.97E-03 | 1.80E-03 |
| 8q24.1 | 28 | 0.85 | 2.00 | 2.00E-04 | 9.77E-04 |
